# An Empirical Torsional Spring Model for the Inclined Crack in a 3D-Printed Acrylonitrile Butadiene Styrene (ABS) Cantilever Beam

**DOI:** 10.3390/polym15030496

**Published:** 2023-01-18

**Authors:** Zhichao Yang, Feiyang He, Muhammad Khan

**Affiliations:** 1 School of Aerospace, Transport and Manufacturing, Cranfield University, College Road, Cranfield MK43 0AL, UK; 2 Centre for Life-Cycle Engineering and Management, School of Aerospace, Transport and Manufacturing, Cranfield University, College Road, Cranfield MK43 0AL, UK

**Keywords:** crack angle, natural frequency, torsional spring stiffness

## Abstract

This paper presents an empirical torsional spring model for the inclined crack on a 3D-printed ABS cantilever beam. The work outlined deals mainly with our previous research about an improved torsional spring model (Khan-He model), which can represent the deep vertical (90°) crack in the structure. This study used an experimental approach to investigate the relationships between the crack angle and torsional spring stiffness. ABS cantilever beams with different crack depths (1, 1.3 and 1.6 mm) and angles (30, 45, 60, 75 and 90°) were manufactured by fused deposition modelling (FDM). The impact tests were performed to obtain the dynamic response of cracked beams. The equivalent spring stiffness was calculated based on the specimen’s fundamental frequency. The results suggested that an increased crack incline angle yielded higher fundamental frequency and vibration amplitude, representing higher spring stiffness. The authors then developed an empirical spring stiffness model for inclined cracks based on the test data. These results extended the Khan-He model’s application from vertical to inclined crack prediction in FDM ABS structures.

## 1. Introduction

Micro-cracks are one of the common damages in structures due to processing flaws during manufacturing. They may propagate under the cyclic loads in the service lifespan and lead to the final fatigue fracture of the structure. Therefore, tiny cracks in structures are critical safety hazards and must be monitored carefully to avoid catastrophic failure [1]. The industry typically uses the Non-Destructive Testing (NDT) method to detect and predict these possible cracks. Among them, the change of structural dynamic response, such as natural frequency and modal response, is a good representation of the damage severity because the crack changes the local stiffness of the structure and thus affects the global vibration behaviours [2,3,4,5].

Comprehensive research has been conducted on this topic in academia. In Ref. [6], the effect of discontinuities on the dynamic response of the structure was discussed. In 1991, Ostachowicz et al. first proposed the torsional spring representation for cracks on the cantilever beam. The change in the local stiffness of the structure due to cracks was analytically modelled and quantitatively converted into the spring stiffness in their research. Both single-sided and double-sided crack cases in the opening mode (first mode of fracture) were analysed and represented by two different torsional spring models [7].

Since the cracks with different depths can be easily replaced by torsional springs with corresponding stiffness, the Ostachowicz model enabled and simplified the modal analysis of the cracked structure. It was then widely used by a large amount of research [8,9,10,11,12]. Radhakrishnan used the Ostachowicz model to simplify a rectangular cantilever beam structure with cracked beams to help him study the effect of crack length on the spring stiffness, the fundamental frequency, vibration amplitude and the occurrence of resonance [8]. In Ref. [9], ALGOR software and the Ostachowicz model was used to analyse the relationship between the intrinsic frequency of the beam structure and the crack depth, as well as the intrinsic frequency of the beam structure and the location of the crack. It is evident from the simulations that when a structure suffers from damage, its dynamic property can change, and it was observed that cracks caused a stiffness reduction with an inherent reduction in natural modal frequencies. In Ref. [10], the Ostachowicz model, LASSO and Ridge regression models were used to help the study determine the cracks’ location and depth by measuring the beam structure’s first three natural frequencies. Ultimately the study also presents a reliable regression model. Loutridis et al. instead attempted to find patterns in the variation of crack size through changes in stiffness and empirical modelling, which is referred to as crack breathing. Based on vibrations, the study analysed the variation of instantaneous frequency with crack depth. The analysis concluded that both the harmonic distortion and the variation of instantaneous frequency increase with crack size, which offers the possibility of calculating crack size from this. The article finally proposed a more efficient vibration-based crack detection technique [11]. Nematollahi et al. chose to use wavelet neural networks (NN; wave-net) and the Ostachowicz model for the identification of crack features. The study was based on simulations and thus obtained complete structural vibration parameters of the beam with the help of finite element analysis, and then cracks at different locations and depths on the beam were introduced to train the wavelet neural network. Ultimately, the identification results are compared with those of some convectional NNs, including radial basis function and multilayer perceptron ones. Results show good accuracy and efficiency of the proposed NN method [12].

Nevertheless, the classical torsional spring model used in the above studies has a certain limitation. It only simulates the light crack and brings a significant dynamic response error when the depth of the crack exceeds a certain percentage of the structural thickness of the structure. Therefore, an improved torsional spring model was proposed by He et al. to reduce the dynamic response error in the case of deep cracks [13].

All the above research only considered the ideal case of a vertical crack. However, the crack cannot be perfectly vertical. An inclined oblique crack is common in reality. It inevitably affects the local stiffness and changes the dynamic response of the structure. Meanwhile, the torsional spring model proposed by Ostachowicz et al. is based on the crack in the opening mode (first mode of fracture). It means the model is only applicable to the ideal vertical crack. It necessarily introduces some errors when representing oblique cracks caused by a mixed-mode fracture (first and second mode of fracture). Several studies have addressed this and presented some outcomes [14,15,16]. In Ref. [17], the shear stiffness loss of the structure with a diagonal crack was determined. In Refs. [18,19], the influence of crack geometries on the vibration characteristics of the spring steel cantilever beam was investigated. They compared the stiffness obtained from V-shaped, U-shaped and rectangular-shaped crack models, and the results suggested that the U-shaped crack was more sensitive. In Ref. [20], the transverse vibration of a slender beam in the presence of an inclined edge crack using a rotational spring was modelled. It potentially enabled crack detection based on the measurement of natural frequencies. The characteristic equation obtained from the vibration analysis of the beam was manipulated to give a relationship between the spring’s stiffness and the crack’s angle. Likewise, Ref. [4] predicted cracks in different orientations in a hollow steel pipe based on the measurement of change in natural frequencies and modelling of cracks by a rotational spring. Moreover, Ref. [21] proposed an analytical model to calculate the natural frequency of a cantilever beam with a breathing-inclined crack based on a double-linear-springs model.

After reviewing the previous context, although these studies above investigated the local stiffness and natural vibrations for structures with inclined cracks, only one of the vibration properties, natural frequencies, was considered. However, the other dynamic behaviours, such as the resonance amplitude and mode shapes, were also affected by the inclined crack. They are other suitable parameters which could represent the flaw conditions appropriately. Furthermore, the manufacturing process also has a significant influence on dynamic response. In particular, fused deposition modelling (FDM) technology provides more complicated mesostructures, affecting the structural stiffness significantly and leading to more unpredictable dynamic responses. Therefore, the inclined crack detection in FDM structures needs to be emphasised compared with the structures made by traditional manufacturing technologies mentioned in previous studies.

The presented study aims to fill the above research gap. It extended the Khan-He models application to inclined crack detection in this work. The paper proposed a modified Khan-He model to provide a more precise representation of the inclined crack scenario. An experimental approach was used to investigate the relationships between the crack angle and torsional spring stiffness. ABS cantilever beams with different crack depths (1, 1.3 and 1.6 mm) and angles (30, 45, 60, 75 and 90°) were manufactured by fused deposition modelling (FDM). The impact tests were performed to obtain the cracked beams’ fundamental frequency and first-order resonance amplitude. The equivalent spring stiffness was calculated based on the specimen’s fundamental frequency. The results suggested that an increased crack incline angle yielded higher fundamental frequency and vibration amplitude, representing higher spring stiffness. The authors then developed an empirical spring stiffness model for inclined cracks based on the test data. These results extended the Khan-He model’s application from vertical to inclined crack prediction in FDM ABS structures.

## 2. Materials and Methods

**Figure 1:**
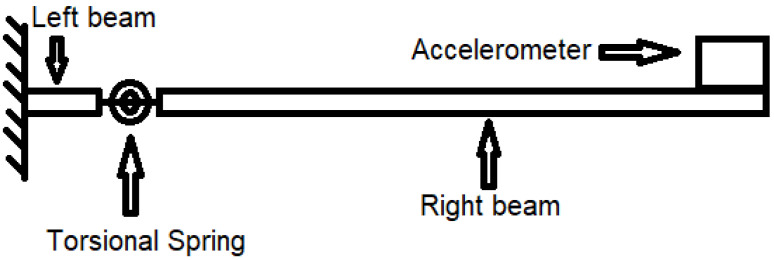
Simulation of a cracked cantilever beam with an accelerometer at beam free end.

This study aims to investigate the vibration behaviour of the cantilever beams with different artificial inclined angle and length cracks. The cracked cantilever beam in the test was modelled as a system shown in Figure 1. It consisted of a torsion spring simulating the crack, two intact beams and an end mass. Two experiments, the impact test and the resonance test, were carried out to measure the system’s dynamic response (fundamental natural frequency and first-order resonance amplitude). This section presents the relevant specimen preparation, experimental scheme, setup and procedures.

### 2.1. Specimen Geometry and Fabrication

**Figure 2:**
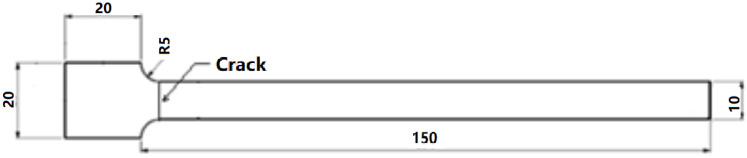
Specimen geometry and dimensions.

The cracked cantilever beam was used as a base in the research due to its easy-to-observe vibration behaviour, which was consistent with our previous studies [13]. The geometry of the specimen is shown in Figure 2. It was 150 mm in length, 10 mm in width with 3 mm in thickness. The crack width was 0.6 mm. In subsequent experiments, there was a 20 mm × 20 mm square at the fixed end for fixing the beam. The default constant distance from the fixed end to the crack was set as 5 mm, which can significantly differentiate dynamic responses between different crack settings.

In order to control crack length and directions precisely, fused deposition modelling technology was applied to fabricate the specimens. The cracked beams with various crack angles and lengths were modelled by CATIA V5R21 and exported as .STL files. The geometry files were then imported into Ultimaker Cura and saved as .GCODE files. Ultimaker 2+ 3D printer read the files and completed the final manufacturing works.

### 2.2. Design of Experiment

Crack angle and crack length were investigated in the research. There were five different crack angles and three different crack lengths, respectively. Therefore, there were 15 different combinations, as shown in Table 1. A 90° inclined angle represented a vertical crack. The minimum value of the angle range was set to 30° to ensure the artificial crack quality. The range of crack length was between 1 mm to 1.6 mm to obtain a stable dynamic response. Each test was repeated three times to ensure the reproducibility of the experiment.

**Figure 3:**
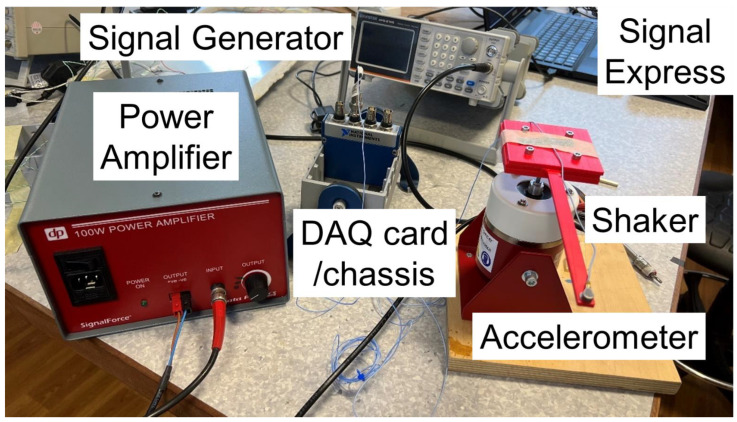
Experimental setups.

Figure 3 shows the experimental setup. The specimen was fixed on a V5 shaker (Data Physics). The signal generator (AFG21105) produced the specified voltage to the power amplifier. Then, the signal amplitude was adjusted and excited the shaker movement. An accelerometer (PCB 352A21) was fixed on the beam free end to record the time and acceleration. DAQ card and chassis transferred the measured data to a Signal Express 2015 in the laptop. The software saved the experimental data as .txt files for subsequent data processing.

**Figure 4:**
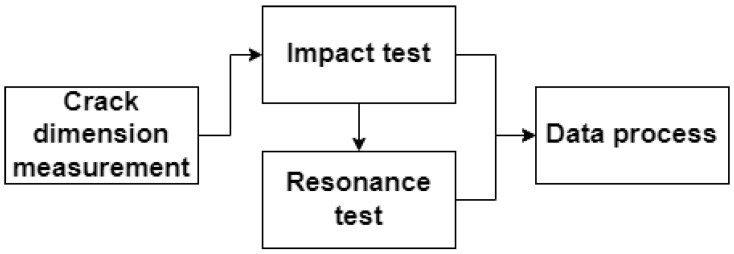
Experimental procedures.

**Figure 5:**
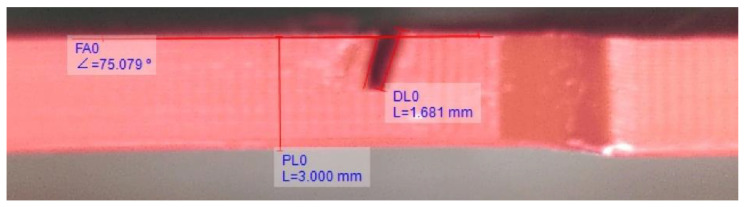
An example of crack dimension measurement by DinoCapture 2.0.

The experimental procedures were divided into four stages, as shown in Figure 4. In the first stage, the dimensions of the artificial crack were measured by a visual approach. A Dino-Lite digital microscope captured the details of the crack. The crack length and inclined angle were measured in DinoCapture 2.0, as shown in Figure 5. Next, the impact test was carried out three times for each specimen to measure the fundamental frequencies and calculate the average value. The obtained mean fundamental frequency was then fed to the signal generator. It drove the shaker to vibrate sinusoidally with a 1 mm amplitude. This frequency excited the beam specimen to resonance. The accelerometer recorded the acceleration amplitude and corresponding time for the following data processing.

### 2.3. Data Processing

The fundamental frequency and first-order resonance amplitude were calculated by Equations (1) and (2), respectively, where $t_{i}$ and $t_{j}$ denoted the time of the ith and jth peak amplitude, $a_{max}$ was the acceleration amplitude during the resonance test.

(1) $$f=\frac{i-j}{t_{i}-t_{j}}$$

(2) $$U=\frac{a_{max}}{{\left(2\pi f\right)}^{2}}$$

The equivalent torsional spring stiffness k was calculated by solving the determinant in Equation (3), where $\beta^{4}=\omega^{2}\rho A/EI$. It was derived from the governing equation of motion for the beam–spring–beam system. Our previous study [13] considered the accelerometer’s mass $m_{acc}$ at the beam end and introduced it into Equation (3), where ω is the fundamental angular frequency, ρ=1050 kg/m^3^ is the density of ABS, A denotes the beam’s cross-section area, E= is the tensile modulus of ABS, I denotes the beam’s moment of inertia, l equals the distance between the crack location and beam’s fixed end and L represents the specimen length.

(3) $$|\begin{array}{cccccccc} 1 & 0 & 1 & 0 & 0 & 0 & 0 & 0\\ 0 & 1 & 0 & 1 & 0 & 0 & 0 & 0\\ \sin(\beta l) & \cos(\beta l) & \sinh(\beta l) & \cosh(\beta l) & -\sin(\beta l) & -\cos(\beta l) & -\sinh(\beta l) & -\cosh(\beta l)\\ -\cos(\beta l) & \sin(\beta l) & -\cosh(\beta l) & -\sinh(\beta l) & \cos\left(\beta l\right)+\frac{E_{T}I}{k}\beta \sin\left(\beta l\right) & -\sin\left(\beta l\right)+\frac{E_{T}I}{k}\beta \cos\left(\beta l\right) & \cosh\left(\beta l_{L}\right)-\frac{E_{T}I}{k}\beta \sinh\left(\beta l_{L}\right) & \sinh\left(\beta l_{L}\right)-\frac{E_{T}I}{k}\beta \cosh\left(\beta l\right)\\ -\sin(\beta l) & -\cos(\beta l) & \sinh(\beta l) & \cosh(\beta l) & \sin(\beta l) & \cos(\beta l) & -\sinh(\beta l) & -\cosh(\beta l)\\ -\cos(\beta l) & \sin(\beta l) & \cosh(\beta l) & \sinh(\beta l) & \cos(\beta l) & -\sin(\beta l) & -\cosh(\beta l) & -\sinh(\beta l)\\ 0 & 0 & 0 & 0 & -\sin(\beta L) & -\cos(\beta L) & \sinh(\beta L) & \cosh(\beta L)\\ 0 & 0 & 0 & 0 & -\cos\left(\beta L\right)+\frac{m_{acc}\beta }{\rho A}\sin\left(\beta L\right) & \sin\left(\beta L\right)+\frac{m_{acc}\beta }{\rho A}\cos\left(\beta L\right) & \cosh\left(\beta L\right)+\frac{m_{acc}\beta }{\rho A}\sinh\left(\beta L\right) & \sinh\left(\beta L\right)+\frac{m_{acc}\beta }{\rho A}\cosh\left(\beta L\right) \end{array}|=0$$

It is worth noting that although the previous study [13] proposed the analytical torsional spring model based on the crack depth and calculated the stiffness k analytically, as shown in Equation (4), where a is the crack length. b and H are beam width and thickness, respectively, $f_{r}\left(\frac{a}{H}\right)$ is the shape function for beam structure, this paper did not use the analytical solution. The reason is that the crack length a in Equation (4) is equal to the crack depth in the case of the vertical crack but not for the oblique crack. Furthermore, the derivation of Equation (4) was based on the stress intensity factor for mode one fracture, which was the same as [7], which was clearly not applicable to inclined cracks in mixed mode fractures. These two aspects led to the inaccuracy of the previous analytical solution in the case of inclined cracks. Therefore, the presented work discarded the previous analytical model and instead attempted to use experimental data to invert the appropriate empirical model.

(4) $$k=\left(\frac{H-a}{H}\right)\times \frac{EbH^{2}}{72\pi f_{r}\left(\frac{a}{H}\right)}$$

The study simply quoted the determinant in Equation (3) and used the experimental fundamental frequency data to calculate equivalent torsional spring stiffness values. In other words, the calculation here was the inverse of our previous study. The result of the calculation did not directly reflect the influence of the crack length and angle. It simply indicated what value the spring stiffness should be for this beam–spring–beam system at a given fundamental frequency.

### 2.4. Modified Khan-He Model

Although Equation (3) is able to give the solution for the equivalent torsional spring stiffness, it is still deficient—i.e., it does not take into account the location of the equivalent spring in the case of inclined cracks. Therefore, this section is dedicated to analysing the relationship between crack angle and spring location, and further the influence on the Khan-He model.

Since only vertical cracks were considered in the original khan-He model, the distance from the crack to the fixed end of the cantilever beam is constant during the crack propagation. It means that the horizontal distance from the crack tip to the fixed end of the beam is the same as the initial position of the cracks (the crack location on the surface of the beam). Therefore, it is straightforward for the value of the distance from the torsional spring to the beam fixed end in the governing equation of motion. In other words, the length of the left-hand beam is fixed in Figure 1 for vertical cracks of any length.

**Figure 6:**
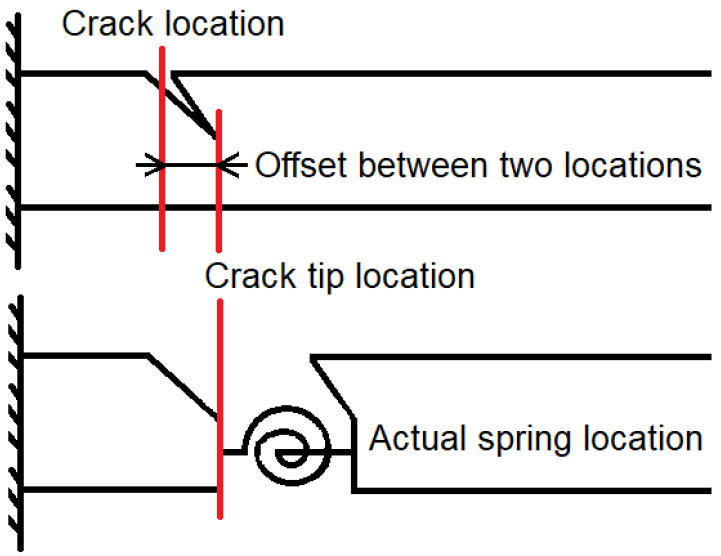
Schematic of the actual crack tip and torsional spring location for inclined crack.

However, the situation has changed for inclined cracks. As shown in Figure 6, since the crack grows at an angle, this means that horizontally the crack tip is positioned gradually away from or close to the fixed end of the beam. Additionally, the distance between the crack tip and the beam’s fixed end must not be equal to the initial situation (the crack at the surface of the beam). There is an offset between them which is different from the vertical crack case.

When the solid structure of a beam with an inclined crack is converted into a beam–spring–beam system, there is, in fact, a connection only at the crack tip location. In other words, the beam on the right is actually moving around the tip of the inclined crack instead of the initial crack location. Likewise, the continuity between the beam on the left and the torsion spring is at the crack tip in the beam–spring–beam system. The reference point of the coordinate system for the motion of the beam on the right is shifted from the initial position of the crack to the crack tip compared to the case of a vertical crack. The horizontal distance of this offset can be expressed in Equation (5), where θ denotes the crack angle.

(5) $$l_{offset}=a\cos\theta$$

Finally, l in Equation (3) was replaced by $l+l_{offset}$. The new determinant was then used to calculate the equivalent torsional spring stiffness values. Since the crack length in the study was only around 1.3 mm, this tiny offset did not significantly affect the calculation results.

## 3. Results and Discussion

There were two independent variables, crack length and crack inclined angle in the experiments. Fundamental frequency, resonance amplitude and equivalent torsional spring stiffness were changed with the various crack dimensions. In order to investigate the potential relationships between them, the crack depth ratio φ was introduced and defined in Equation (6). It represents the depth percentage of the beam occupied by the crack.

(6) $$\varphi =\frac{a\sin\theta }{H}$$

The correlation analysis between these variables was carried out to investigate their interdependencies. The research calculated the Pearson correlation coefficient r (level of significance *p* < 0.05), which was used to quantify the influence level of the crack dimension on dynamic response and the spring stiffness. The results are exhibited in Table 2.

### 3.1. Effect of Crack Length on the Dynamic Response

As this paper underlines the crack angle’s influence, the crack size’s influence on the beam’s dynamic response will be discussed briefly here. A large number of studies have investigated the vibration behaviour of cracked structures by analytical, numerical or empirical approaches [13,22,23]. They all suggested that a larger crack length reduced both the fundamental frequency and first-order resonance amplitude.

The same trends were exhibited in our experimental results. The *p* values for the correlation between crack length and dynamic response were lower than 0.05 in Table 2. It suggested that there were significant dependencies between them. The negative r values for f and U implied that increased crack length was responsible for a shift to a lower fundamental frequency and resonance amplitude. The argument corroborated the conclusion of previous studies. The underlying cause of the different vibration behaviours was the change in local structural stiffness at the crack area. Longer cracks cut off more ABS fibres. Thus, it reduces the stiffness and leads to a smaller dynamic response.

### 3.2. Effect of Crack Dimension on Equivalent Torsional Spring Stiffness

**Figure 7:**
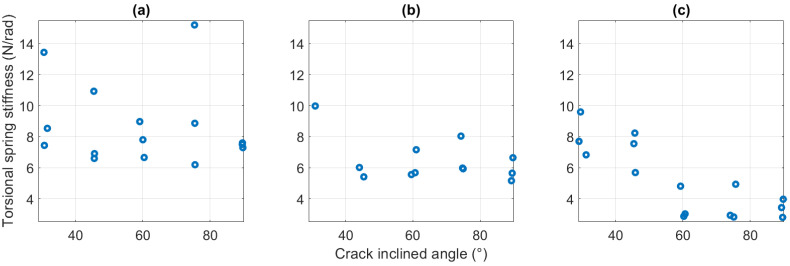
Equivalent torsional spring stiffness for inclined cracks with different angles. (**a**) 1 mm crack length. (**b**) 1.3 mm crack length. (**c**) 1.6 mm crack length.

The *p*-value 0.0695 provided by correlation analysis (Table 2) appeared to suggest no significant influence of the crack angles on the spring stiffness. However, the corresponding r value −0.2731 and Figure 7 demonstrated that the increased inclined angle resulted in lower spring stiffness. Furthermore, compared to Figure 7a,b, Figure 7c showed a more significantly reduced trend from around 8 N/rad of 30° inclined angle to 3.5 N/rad of 90° inclined angle, which attained a reduction in stiffness of over 50%.

This notable effect of the crack angle on spring stiffness has similar theoretical support as the crack length influence. For cracks of constant length, the number of ABS filaments severed by a vertical crack (90°) must be the highest in cross-section. It results in the poorest local structural stiffness. The number of fractured ABS chains gradually decreases as the cracks tilt, enhancing the structural strength. Thus, it is modelled with higher torsional stiffness. As for the distinction of the trend for 1.6 mm crack length, it will break more of the ABS chain when the crack inclined angles are all changed from 30° to 90° when compared with 1- and 1.3 mm cracks. In other words, the crack depth ratio φ of 1.6 mm crack has a more significant change in this case, so it yields a steeper negative trend in Figure 7c.

### 3.3. Effect of Crack Angle on the Dynamic Response

Section 3.2 has pointed out the significant effect of the crack-inclined angle on the local stiffness. Therefore, theoretically, the fundamental frequency and resonance amplitude, which are highly dependent on the beam’s properties, could also be affected by the crack angle. *p* values (0.0010 for fundamental frequency and 0.0383 for resonance amplitude) in Table 2 confirmed their significant dependencies. Additionally, the negative r values indicate that both the fundamental frequency and resonance amplitude have the same decreased trends when the crack is close to the vertical position.

**Figure 8:**
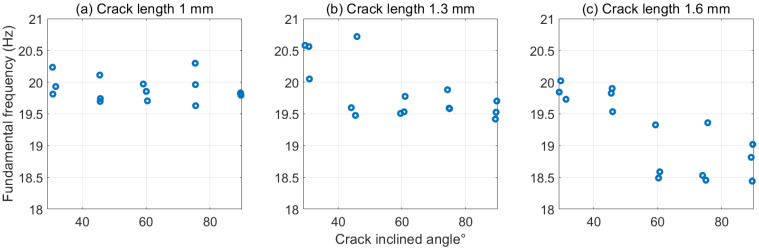
Fundamental frequencies for inclined cracks with different angles. (**a**) 1 mm crack length. (**b**) 1.3 mm crack length. (**c**) 1.6 mm crack length.

**Figure 9:**
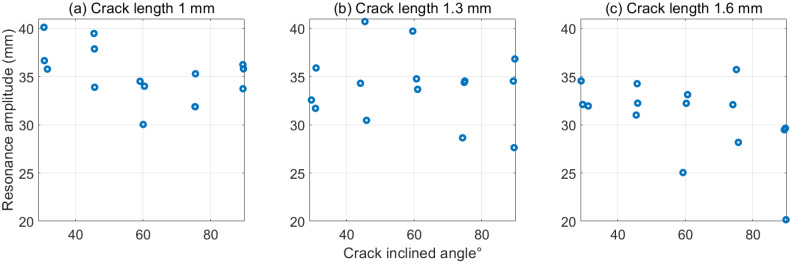
First-order resonance amplitude for inclined cracks with different angles. (**a**) 1 mm crack length. (**b**) 1.3 mm crack length. (**c**) 1.6 mm crack length.

Similarly, Figure 8 and Figure 9 demonstrate the same lower values of dynamic response with the increased crack-inclined angle. Although the distribution of the experimental data points is dispersed because of subtle differences within the structures caused by FDM, the lower fundamental frequencies and smaller resonance amplitudes were observed when increasing the crack angle. This trend was particularly noticeable when the cracks were relatively deep. Figure 7c shows that the frequency drops from around 19.8 to 18.7 Hz for a 1.6 mm depth crack with an increased angle. Likewise, an average 25 mm resonance amplitude for a 90° crack is significantly lower than approximately 32 mm for a 30° crack in Figure 9c. The reasons behind this have already been mentioned in Section 3.2. Another important point to keep in mind is that compared with the fundamental frequency, the resonance amplitude responses in Figure 9 were more random than in Figure 8. The three values of data from the repeated experiments were also more dispersed in Figure 9. The reason for this may be due to the fact that the noise and disturbance from the accelerometer’s wiring vibration during the resonance test.

### 3.4. Regression Modelling between the Inclined Crack and Torsional Spring Stiffness

As a result of the discussion in Section 3.2 and Section 3.3, one crucial point to keep in mind is that the local stiffness in the oblique crack area and the beam’s dynamic response significantly depend on the number of breaking ABS filaments. Coincidentally, this parameter can be quantified in relation to φ the crack depth ratio directly. This conclusion is consistent with the super small *p*-values between φ, f and U in Table 2.

**Figure 10:**
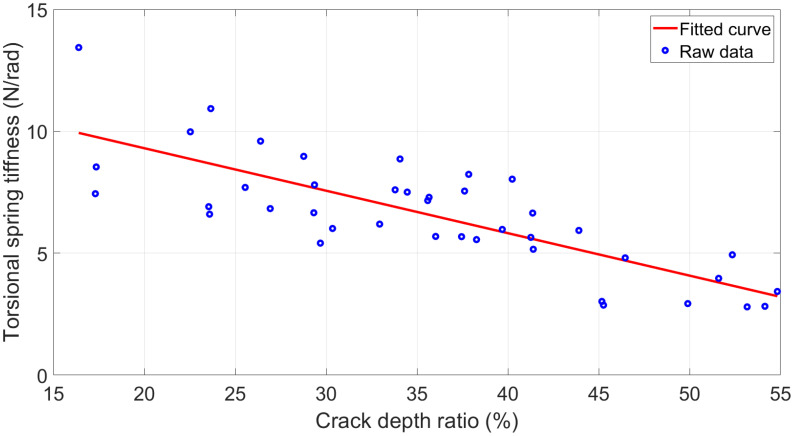
Regression model for torsional spring stiffness.

Therefore, a regression model between the crack depth ratio φ and equivalent torsional spring stiffness k was developed, as shown in Equation (7) and Figure 10, with a 62.76% R-square value.

(7) k=−0.1743φ+12.79

**Figure 11:**
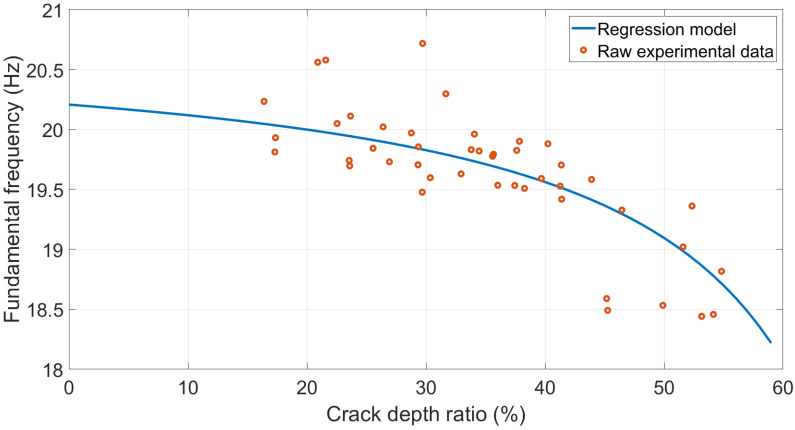
Modelled fundamental frequency and experimental data comparison.

The modelled stiffness was then substituted into the determinant in Equation (3) to calculate the modelled fundamental frequency for validation. The results are shown in Figure 11. The experimental fundamental frequency data points were distributed along the regression curve. The maximum difference between the raw data and the regression model was around 4.3%, which was acceptable.

## 4. Conclusions

The present research aimed to determine the potential dependencies between the oblique crack and the local structural stiffness, which a rotational spring model simulated. An empirical relationship was proposed and validated between the stiffness and crack depth ratio derived from crack length and angle. This empirical model extended the Khan-He model’s application from the vertical to the inclined crack scenario.

The FDM ABS cantilever beams with different inclined cracks were tested to investigate their dynamic response and determine the simulated torsional spring stiffness. The experiments confirmed that a larger crack length reduced the fundamental frequency and first-order resonance amplitude. Furthermore, the results suggested that the increased crack angle reduced the torsional spring stiffness and structural dynamic response. The reason behind this was attributed to the number of ABS filaments cut in the FDM structure. The findings contributed to the physical understanding of the inclined crack influence on FDM polymeric structures.

## Figures and Tables

**Table 1:** Crack parameter combination.

Test No.	Inclined Angle (°)	Crack Length (mm)
1	90	1
2		1.3
3		1.6
4	75	1
5		1.3
6		1.6
7	60	1
8		1.3
9		1.6
10	45	1
11		1.3
12		1.6
13	30	1
14		1.3
15		1.6

**Table 2:** Correlation analysis between crack dimension, dynamic response and spring stiffness (dependent variables were marked with yellow).

	Crack Length a	Crack Inclined Angle θ	Crack Depth Ratio φ	Fundamental Frequency f	Resonance Amplitude U	Torsional Spring Stiffness k
a	1					
θ	0.0243 (*p* = 0.8739)	1				
φ	0.6390 (*p*= 2.3079 × 10^−6^)	0.7446 (*p* = 4.5028 × 10^−9^)	1			
f	−0.5649 (*p* = 5.2835 × 10^−5^)	−0.4730 (*p* = 0.0010)	−0.7768 (*p* = 3.5597 × 10^−10^)	1		
U	−0.4506 (*p* = 0.0019)	−0.3100 (*p* = 0.0383)	−0.4981 (*p* = 4.9826 × 10^−4^)	0.2181 (*p* = 0.1500)	1	
k	−0.1330 (*p* = 0.3838)	−0.2731 (*p* = 0.0695)	−0.2931 (*p* = 0.0507)	0.5554 (*p* = 7.4999 × 10^−5^)	−0.0747 (*p* = 0.6258)	1

## Data Availability

Data presented in this study are available on request from the corresponding author.

## References

[1] Chen X.F., He Z.J., Xiang J.W. (2005). Experiments on Crack Identification in Cantilever Beams. Exp. Mech..

[2] Zhang K., Yan X. (2017). Multi-Cracks Identification Method for Cantilever Beam Structure with Variable Cross-Sections Based on Measured Natural Frequency Changes. J. Sound Vib..

[3] Barad K.H., Sharma D.S., Vyas V. (2013). Crack Detection in Cantilever Beam by Frequency Based Method. Procedia Eng..

[4] Naniwadekar M.R., Naik S.S., Maiti S.K. (2008). On Prediction of Crack in Different Orientations in Pipe Using Frequency Based Approach. Mech. Syst. Signal Process..

[5] Douka E., Hadjileontiadis L.J. (2005). Time-Frequency Analysis of the Free Vibration Response of a Beam with a Breathing Crack. NDT E Int..

[6] Kirmsher P.G. (1944). The Effect of Discontinuities on the Natural Frequency of Beams. Proc. ASTM.

[7] Ostachowicz W.M., Krawczuk M. (1991). Analysis of The Effect of Cracks on The Natural Frequencies of a Cantilever Beam. J. Sound Vib..

[8] Radhakrishnan V.M. (2004). Response of a Cracked Cantilever Beam to Free and Forced Vibrations. Def. Sci. J..

[9] Sutar M.K., Pattnaik S. (2010). Vibration Characteristics of a Cracked Cantilever Beam under Free Vibration. Noise Vib. Worldw..

[10] Choudhury S., Thatoi D.N., Hota J., Sau S., Rao M.D. (2019). Predicting Crack in a Beam-like Structure through an over Fitting Verified Regression Model. Multidiscip. Model. Mater. Struct..

[11] Loutridis S., Douka E., Hadjileontiadis L.J. (2005). Forced Vibration Behaviour and Crack Detection of Cracked Beams Using Instantaneous Frequency. NDT E Int..

[12] Nematollahi M.A., Farid M., Hematiyan M.R., Safavi A.A. (2012). Crack Detection in Beam-like Structures Using a Wavelet-Based Neural Network. Proc. Inst. Mech. Eng. G J. Aerosp. Eng..

[13] He F., Khan M., Aldosari S. (2022). Interdependencies between Dynamic Response and Crack Growth in a 3D-Printed Acrylonitrile Butadiene Styrene (ABS) Cantilever Beam under Thermo-Mechanical Loads. Polymers.

[14] Hajhosseini M., Nahvi H. (2011). Identification of Slant Cracks in a Cantilever Beam Using Design of Experiment and Neuro-Genetic Technique. Key Eng. Mater..

[15] Ismail R., Cartmell M.P. (2012). An Analysis of the Effects of the Orientation Angle of a Surface Crack on the Vibration of an Isotropic Plate. J. Phys. Conf. Ser..

[16] Pansare S.R., Naik S.S. (2019). Detection of Inclined Edge Crack in Prismatic Beam Using Static Deflection Measurements. Sādhanā.

[17] Liu J., Jia Y., Zhang G., Wang J. (2018). Effect of Diagonal Cracks on Shear Stiffness of Pre-Stressed Concrete Beam. Int. J. Struct. Integr..

[18] Khalkar V., Ramachandran S. (2018). The Effect of Crack Geometry on Stiffness of Spring Steel Cantilever Beam. J. Low Freq. Noise Vib. Act. Control.

[19] Khalkar V., Ramachandran S. (2019). The Effect of Crack Geometry on Non-Destructive Fault Detection of EN 8 and EN 47 Cracked Cantilever Beam. Noise Vib. Worldw..

[20] Nandwana B.P., Maiti S.K. (1997). Modelling of Vibration of Beam in Presence of Inclined Edge or Internal Crack for Its Possible Detection Based on Frequency Measurements. Eng. Fract. Mech..

[21] Ma Y., Chen G. (2017). Natural Vibration of a Beam with a Breathing Oblique Crack. Shock Vib..

[22] He F., Thakur V.K., Khan M. (2021). Evolution and New Horizons in Modeling Crack Mechanics of 3D Printing Polymeric Structures. Mater. Today Chem..

[23] He F., Khan M.A., Zai B.A. (2019). Material Mechanics of Crack Growth in Structural Dynamics. Procedia Struct. Integr..

